# Development and Infectious Disease in Hosts with Complex Life Cycles

**DOI:** 10.1371/journal.pone.0060920

**Published:** 2013-04-02

**Authors:** Catherine L. Searle, Gisselle Yang Xie, Andrew R. Blaustein

**Affiliations:** 1 Department of Ecology and Evolutionary Biology, University of Michigan, Ann Arbor, Michigan, United States of America; 2 Department of Zoology, Oregon State University, Corvallis, Oregon, United States of America; 3 Environmental Sciences Graduate Program, Oregon State University, Corvallis, Oregon, United States of America; University of Helsinki, Finland

## Abstract

Metamorphosis is often characterized by profound changes in morphology and physiology that can affect the dynamics of species interactions. For example, the interaction between a pathogen and its host may differ depending on the life stage of the host or pathogen. One pathogen that infects hosts with complex life cycles is the emerging fungal pathogen of amphibians, *Batrachochytrium dendrobatidis* (Bd). We sought to determine how conditions at the larval stage can affect variation in development and patterns of Bd infection across amphibian life stages. We used outdoor experimental mesocosms to simulate natural pond habitats and manipulated the presence of Bd, the larval density, and the number of host species in larvae of two co-occurring amphibian species (*Rana cascadae* and *Pseudacris regilla*). We found that infection differed between species throughout development; *P. regilla* consistently had higher infection severity compared to *R. cascadae*. Additionally, while up to 100% of larvae were infected, only 18.2% of *R. cascadae* and 81.5% of *P. regilla* were infected after metamorphosis. This indicates that amphibians have the ability to recover from Bd infection as they undergo metamorphosis. Higher larval densities in *P. regilla* led to a shorter larval period, and individuals with a shorter larval period had lower infection severity. This led to a trend where *P. regilla* larvae reared at high densities tended to have lower infection prevalence after metamorphosis. We also found that exposure to Bd increased larval mortality and prolonged the larval period in *P. regilla*, indicating that *P. regilla* are susceptible to the negative effects of Bd as larvae. This study demonstrates that host density, species composition, and pathogen exposure may all interact to influence development and infection in hosts with complex life cycles.

## Introduction

Host-pathogen interactions can be influenced by both the genotype and phenotype of the host. For example, genetic resistance to pathogens commonly varies within a host population, with some host genotypes more resistant to a pathogen than others [1]–[3]. Additionally, the physiological and morphological condition of a host can vary with developmental stage, size, and age, and also affect disease risk [4], [5]. Particularly dramatic changes to host physiology and morphology occur in species with complex life cycles, such as those that undergo metamorphosis (e.g. amphibians, holometabolous insects, parasites, marine invertebrates and some fishes). Therefore, as a host undergoes metamorphosis, infectious disease dynamics may vary from one life stage to another. The life stage of an organism can affect disease risk, transmission and susceptibility [6], [7].

One pathogen that infects species with complex life histories is the fungus, *Batrachochytrium dendrobatidis* (Bd), which has been associated with population declines and extinctions of amphibians around the world [8]–[11]. The infectious stage of Bd is the motile, waterborne zoospore [12]. Contact with zoospores in water is a key mechanism for Bd transmission to an amphibian host [13]–[15], although direct contact between susceptible and infected individuals may also lead to transmission [16]. Both larval and post-metamorphic amphibians can shed zoospores that can infect new hosts [13], [14].

Most amphibians have a fully aquatic larval stage and semi-aquatic or terrestrial life stage after metamorphosis. This transition from the larval to post-metamorphic stages creates major physiological changes which potentially can affect Bd infection. Bd infects the keratinized structures of amphibians, which are present in the mouthparts of early-stage larvae and the epidermis of adults [17]. Due to these different locations on the host, the outcome of infection can differ at these two developmental stages. Generally, larval Bd infection is less severe than in post-metamorphic stages, although it can cause lethal and sublethal effects in larvae in some species [13], [18]–[20]. After metamorphosis, infection commonly leads to inappetence, weight loss, lethargy, and death [21]–[23], but some species may be relatively tolerant of post-metamorphic infection [24]–[26]. As a larva undergoes metamorphosis, it loses keratinized mouthparts [17] and may shed infection. However, during the late larval stages, keratin may be present on both mouthparts and regions of the skin at the same time [17]. Therefore, to maintain infection through metamorphosis, a late-stage larvae must develop infection on its skin before it completes metamorphosis. This can occur through self-infection as zoospores travel from infected mouthparts to their newly-keratinized skin. Alternatively, a late-stage larva may become infected on its skin through transmission from other infected individuals [14], [27].

Numerous factors may affect transmission of Bd during metamorphosis [15], [16], [28]–[30]. For example, in many host-pathogen systems, the density of infected conspecifics can influence the likelihood of acquiring infection [15]. Additionally, the presence of reservoir species can amplify infection in non-reservoir species through pathogen-spillover [31], [32]. In hosts with complex life cycles, whether or not a species acts as a reservoir may depend on its life stage. For example, Mountain yellow-legged frogs (*Rana muscosa*) act as reservoirs for Bd as larvae, while the post-metamorphic stage of this species suffers high rates of mortality when infected [33], [34].

While density and species combination may affect infectious disease in amphibians, they can also influence development and metamorphosis. The effects of density on larval period and size at metamorphosis are context-dependent. Generally, higher larval densities will slow development and lead to smaller individuals [35]–[37]. However, when controlling for growth rates, higher larval densities can actually speed metamorphosis if food is not limiting [38]. The presence of other species during the larval period can also influence development. For example, growth and development are often slowed when a superior competitor is present in the larval environment [39]–[41]. The presence of a pathogen can also influence these intra- and interspecific competitive interactions [13], [42], [43]. Therefore, the density of conspecifics, the presence of other species, and the presence of infectious agents may interact in complex and multi-faceted ways. In this study, we used outdoor experimental mesocosms to manipulate larval density, species combination and the presence of Bd to determine how these factors affect metamorphosis and infection across life stages.

## Materials and Methods

### Study organisms

The Cascades frog (*Rana cascadae*) and the Pacific treefrog (*Psuedacris regilla*) are two common syntopic species found in ponds and lakes in the Cascade Mountains, Oregon, USA [44]. Bd has been detected on field-collected individuals of both species [45], [46], but they differ in their patterns of infection. For example, *R. cascadae* larvae exposed to Bd carry lower pathogen loads compared to *P. regilla* larvae exposed to the same number of Bd zoospores [29], [47]. However, despite lower infection severity, *R. cascadae* larvae suffer mouthpart deformities when infected with Bd, while *P. regilla* appear to be unaffected [18]. Due to high infection loads and low Bd-associated mortality, it has been suggested that *P. regilla* may act as reservoirs for Bd [48], [49].

To ensure that animals used in our experiment were not previously infected with Bd, *R. cascadae* and *P. regilla* were collected as eggs from sites in the Cascade Mountains of Oregon, USA (Linn County; elevation ∼1100 m). Eggs were brought to the laboratory where they were kept in dechlorinated water at 13.5–15.0°C with a light regime that mimics outdoor conditions. Upon hatching, larvae were fed a 3∶1 mixture (by volume) of rabbit chow to fish food (Tetramin). Animals were between Gosner stages 26–30 [50] upon initiation of the experiment and had been hatched for approximately 45 days. This study was conducted in accordance with Oregon State University's Institutional Animal Care and Use Committee (permit #3553) and collection permits were obtained from Oregon Department of Fish and Wildlife. Great care was used to reduce suffering. Upon termination of the experiment, any remaining animals were euthanized by overdose of buffered MS-222 as recommended and approved by Oregon State University's Animal Care and Use Committee.

### Experimental design

Bd cultures were maintained in the laboratory in 1% tryptone broth. For use in this experiment, Bd (strain JEL 274 originally isolated from an *Anaxyrus boreas* in Colorado [51]) was cultured onto 1% tryptone agar Petri dishes and allowed to grow for 7–10 days at 22°C. To harvest Bd, Petri dishes were flooded with 15 mL water for 20 minutes to allow zoospores to release. The water from 25 dishes was combined and aliquotted among exposure containers, with some inoculum reserved for zoospore quantification with a hemocytometer (Hausser Scientific). Larvae were exposed to Bd twice during the experiment; once before being moved into the mesocosms and once in the mesocosms. For the pre-mesocosm exposure, 224 larvae of each species were distributed among 8–11 L aquaria for each species (28 larvae per aquaria). For each species, half the aquaria were exposed to Bd at a concentration of approximately 4.4×10^3^ zoospores/mL in their aquaria, and the others exposed to a sham inoculation (control) using sterile agar Petri dishes. Larvae were left in their exposure aquaria for seven days before moving into the mesocosms. This first exposure served to ensure that all animals were exposed to a similar dose of Bd before the start of the experiment, and therefore all had a similar chance of acquiring initial infections, regardless of their treatment.

The number of species and initial density of larval amphibians were experimentally manipulated in outdoor mesocosms that simulated natural bodies of water. The experiment was a 5×2 factorial design with five density/species combinations; (1) five *P. regilla* larvae, (2) 10 *P. regilla* larvae, (3) five *R. cascadae* larvae, (4) 10 *R. cascadae* larvae, and (5) five larvae of each species. Each density/species treatment was either exposed to Bd or unexposed (control) and each treatment was replicated five times for a total of 50 mesocosms.

Mesocosms were established 15 days before initiation of the experiment to allow algal and zooplankton communities to establish. Each plastic mesocosm (94 cm L ×70 cm W ×33 cm H) was filled with 5 g dried oak leaves (*Quercus sp*.), 9 g dried alfalfa pellets, 15 mL concentrated zooplankton suspension (*Daphnia sp*.), and 120 L well water. Therefore, the initial density of tadpoles in the mesocosms was either 0.0833 larvae L^−1^ for the high density treatments or 0.0417 larvae L^−1^ for the low density treatments. These densities are within the natural ranges of amphibian larvae in the Cascade Mountains [52], [53]. Mesocosms were covered with mesh to prevent colonization of predators or escape of our samples. Larvae were randomly assigned to a treatment and mesocosm. After 12 days in the mesocosms, larvae were exposed to Bd for a second time. The same methods as in the laboratory exposure were used to create the inoculum, and the exposure concentration was approximately 62.5 zoospores/mL in the Bd-exposed mesocosms. Control mesocosms were exposed to an inoculum without Bd in the broth. During the experiment, temperatures in the mesocosms ranged from 4.5–22.5°C.

Mesocosms were monitored daily for animals completing metamorphosis. Any animal that completed metamorphosis (designated as full tail resorption; Gosner stage 46 [50]), was removed from the mesocosm and brought to the laboratory for monitoring and to allow post-metamorphic infections to develop. We defined “larval period” for each animal as the number of days from the start of the experiment to the day the animal completed metamorphosis. Post-metamorphic animals were held in individual Petri dishes (140×30 mm) containing a moist paper towel with holes in the lid to facilitate air flow, and fed three pinhead-sized crickets every two days. Fifteen days after metamorphosis, each animal was euthanized with an overdose of buffered MS-222 and preserved in 95% ethanol. Three months after the start of the experiment, any animals remaining in the mesocosms were euthanized and preserved. We thoroughly searched for remaining animals to ensure all surviving animals were collected. Any animal that was unaccounted for at the end of the experiment was considered to have died before metamorphosis.

### Infection analyses

Infection was monitored at three time points during the experiment; 1) after laboratory infection but before larvae were moved into mesocosms, 2) when larvae were in the mesocosms and 3) after animals completed metamorphosis. For time point 1, seven Bd-exposed and three unexposed animals of each species were euthanized and preserved seven days after laboratory exposure. The purpose of testing infection at time point 1 was to ensure that our initial Bd-exposure was successful. For time point 2, the mouthparts of two haphazardly chosen larvae from each mesocosm were swabbed 22 days after addition into mesocosms using a fine-tip sterile swab (Medical Wire and Equipment, UK). The swab was inserted into the mouth of each sampled larvae and rotated for approximately five seconds. For mesocosms that contained two species, an individual of each species was swabbed. The purpose of monitoring infection at time point 2 was to determine if larvae were maintaining their infections in the mesocosms. Finally, for time point 3, all Bd-exposed post-metamorphic animals were swabbed 10 times on their ventral side immediately before euthanization (after 15 days of post-metamorphic monitoring). Therefore, 85 post-metamorphic Bd-exposed animals were tested (44 *R. cascadae* and 41 *P. regilla*) and nine randomly chosen control (unexposed) individuals. The purpose of monitoring infection after metamorphosis was to determine how our treatments at the larval stages affected post-metamorphic infection.

Infection status and severity were analyzed using quantitative polymerase chain reaction (qPCR [54]). DNA was extracted from swabs or mouthparts; whole mouthparts were used for animals at time point 1 while swabs were used on time points 2 and 3. Extraction methods follow Boyle et al. [54] except using 60 µL Prepman Ultra instead of 40 µL (Applied Biosystems®, Life Technologies). QPCR was conducted on an ABI PRISM 7500 (Applied Biosystems) using primers and probe developed by Boyle et al. [54] with each sample analyzed in triplicate to determine the average number of genome equivalents for each individual. We included a no-template control (nanopure water instead of amphibian sample) in each qPCR plate and never observed amplification in these controls. If only one of three replicates tested positive, the sample was re-analyzed. An individual was considered Bd-positive if at least two of three qPCR replicates (run once) or three of six replicates (run twice) were positive. For all Bd-positive individuals, infection severity was calculated as the average number of Bd genome equivalents from the positive qPCR replicates.

### Statistical analyses

Infection severity between species was compared at each time point. Because different methods were used to sample at each time point (whole mouthparts for laboratory larvae, oral swabs for mesocosm larvae and ventral swabs for post-metamorphic animals) we did not statistically compare infections through time. Due to unequal variance, infection severity between species was compared at each time point using Welch's t-tests. Only individuals that tested positive for infection were included in our infection severity analyses. We compared pre-metamorphic survival and post-metamorphic infection prevalence (the proportion infected from each mesocosm) between species using quasibinomial generalized linear models (GLM).

To compare infection prevalence among treatments after metamorphosis, the proportion of post-metamorphic animals that were infected from each mesocosm was analyzed using a binomial GLM, with separate analyses for each species. Additionally, a linear mixed effects model was used on log-transformed post-metamorphic infection severity in *P. regilla* using only animals that tested positive for infection. Our model for *P. regilla* infection severity included species/density treatment, mass at metamorphosis and the length of larval period with individuals nested within mesocosms. Due to low numbers of infected post-metamorphic *R. cascadae* (only 8 infected individuals), it was not possible to statistically compare *R. cascadae* infection severity among treatments.

The proportion of animals experiencing mortality before metamorphosis and proportion completing metamorphosis in each mesocosm was compared using GLM's (one for each species) with Bd-exposure and species combination as predictors. Only one individual died during post-metamorphic monitoring, so post-metamorphic infection severity was not analyzed. The effects of Bd exposure and species combination on mass at metamorphosis and larval period were each analyzed using a linear mixed effects model with individuals nested by mesocosm. Animals not completing metamorphosis were excluded from the mass and larval period analyses. All statistics were performed in R version 2.15.1 [55].

## Results

**Figure 1:**
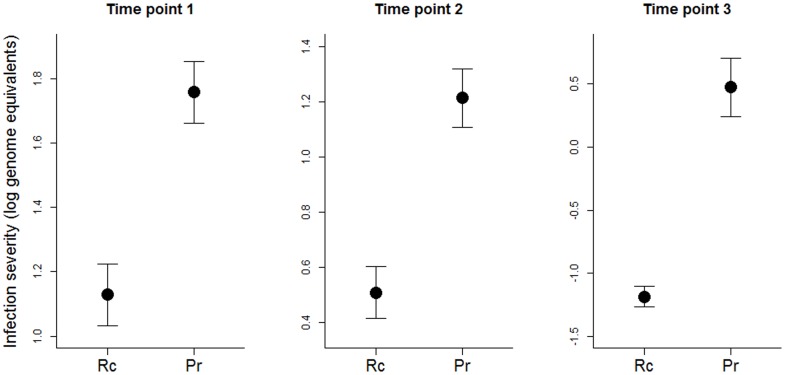
Average infection severity in *Rana cascadae* (“Rc”) was lower than *Pseudacris regilla* (“Pr”) at all time points (±SE). Whole larval mouthparts were used for time point 1 (after laboratory infection), oral swabs for larvae at time point 2 (when animals were in the mesocosms) and skin swabs for time point 3 (after metamorphosis). Only animals that tested positive for infection are shown.

Upon termination of the experiment 38% of animals across all treatments had completed metamorphosis, 26% died and 36% were euthanized (were still larvae). In all, 72 *P. regilla* (41 Bd-exposed and 31 unexposed) and 80 *R. cascadae* completed metamorphosis (44 Bd-exposed and 36 unexposed). Pre-metamorphic survival was lower in *P. regilla* compared to *R. cascadae* (F_1,58_ = 5.75, p = 0.020). At all time points, infection severity was lower in *R. cascadae* than *P. regilla* (time point 1: t = 4.63, p = 0.001; time point 2: t = 4.96, p<0.001, time point 3: t = 7.22, p<0.001; Fig. 1). All Bd-exposed animals that were tested for infection at time point 1 were infected (7 *R. cascadae* and 7 *P. regilla*). At time point 2 we detected Bd in 80% (20 of 25) of *R. cascadae* larvae and 96% (24 of 25) of *P. regilla* larvae exposed to Bd using oral swabs. Since oral swabs are unable to detect low levels of Bd infection [56], it is possible that a greater percentage of the larvae at time point 2 were infected with Bd. After metamorphosis (time point 3) only 18.18% (8 of 44) of Bd-exposed *R. cascadae* were positive for infection, while 81.49% (33 of 41) of *P. regilla*, exposed to Bd tested positive for Bd. A higher proportion of post-metamorphic *R. cascadae* were infected compared to *P. regilla* (F_1,25_ = 18.87, p<0.001). All of our control (unexposed) animals tested negative for infection at all time points.

**Figure 2:**
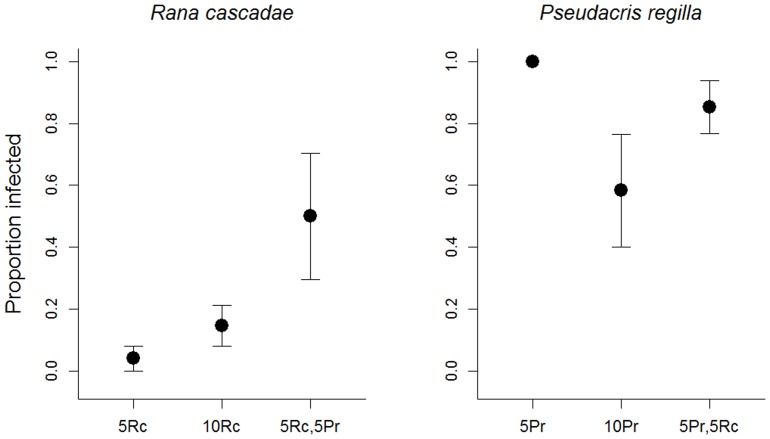
Post-metamorphic infection prevalence in *Rana cascadae* and *Pseudacris regilla* for mesocosms in each treatment (±SE). Treatments are indicated on the x-axis with the number of individuals indicated as “Rc” for *R. cascadae* and “Pr” for *P. regilla*. In the 5Pr treatment, all mesocosms experienced 100% infection.

**Figure 3:**
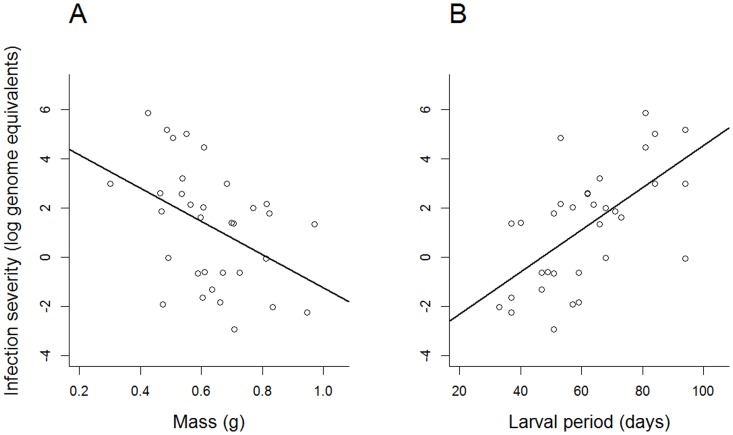
Relationship between A) mass at metamorphosis and B) larval period on log-transformed post-metamorphic infection severity in *Pseudacris regilla*. Only animals that tested positive for infection are included.

In both *P. regilla* and *R. cascadae*, there was a trend for density/species combination treatment to affect infection prevalence (*R. cascadae*: *X^2^* = 5.27, p = 0.072; *P. regilla*: *X^2^* = 4.96, p = 0.084; Fig. 2, Table 1). In *R. cascadae*, mesocosms with five *R. cascadae* had the lowest infection prevalence, mesocosms with 10 *R. cascadae* intermediate and the two species combination had the highest infection prevalence (Fig. 2). In *P. regilla*, mesocosms with only five *P. regilla* had the highest infection prevalence while mesocosms with 10 *P. regilla* had the lowest (Fig. 2). Infection severity in *P. regilla* was not affected by treatment (F_2,9_ = 2.82, p = 0.112; Table 1). Nevertheless, heavier *P. regilla* and those that completed metamorphosis sooner had lower infection severity (mass: F_1,19_ = 7.38, p = 0.014, r^2^ = 0.181; larval period: F_1,19_ = 16.37, p = 0.001, r^2^ = 0.379; Fig. 3, Table 1).

**Table 1:** Summary of statistical findings.

Species	Predictor	Dependent variable	Results	Pattern
*Rana cascadae*	Density and species treatment	Post-metamorphic infection prevalence^1^	*X* 2 = 5.27, p = 0.072	Trend for higher infection prevalence when *P. regilla* present
		Pre-metamorphic survival^2^	*X* 2 = 3.31, p = 0.209	-
		Larval period^3^	F = 1.47, p = 0.251	-
	Bd exposure	Pre-metamorphic survival^2^	*X* 2 = 0.50, p = 0.479	-
		Larval period^3^	F = 0.04, p = 0.851	-
*Pseudacris regilla*	Density and species treatment	Post-metamorphic infection prevalence^1^	*X* 2 = 4.96, p = 0.084	Trend for higher infection prevalence at low densities
		Post-metamorphic infection severity^4^	F = 2.82, p = 0.112	-
		Pre-metamorphic survival^2^	*X* 2 = 0.80, p = 0.669	-
		Larval period^3^	F = 5.10, **p = 0.017**	Longer larval period at low densities
	Mass at metamorphosis	Post-metamorphic infection severity^4^	F = 7.38, **p = 0.014**	Lower infection severity with increased mass
	Larval period	Post-metamorphic infection severity^4^	F = 16.37, **p = 0.001**	Lower infection severity with shorter larval period
	Bd exposure	Pre-metamorphic survival^2^	*X* 2 = 5.86, **p = 0.016**	Reduced survival with Bd exposure
		Larval period^3^	F = 5.56, **p = 0.029**	Longer larval period with Bd exposure

1 Post-metamorphic infection prevalence was analyzed using a binomial GLM with the density and species treatments as predictors.

2 Proportion of animals in each mesocosm surviving to metamorphosis was analyzed using a binomial GLM with Bd treatment and density and species treatments as predictors.

3 Larval period was analyzed using a mixed effects model with Bd treatment and density and species treatments as predictors with individuals nested by mesocosm.

4 Post-metamorphic infection severity was analyzed using a mixed effects model with density and species treatments, mass at metamorphosis and larval period as predictors with individuals nested by mesocosm.

**Figure 4:**
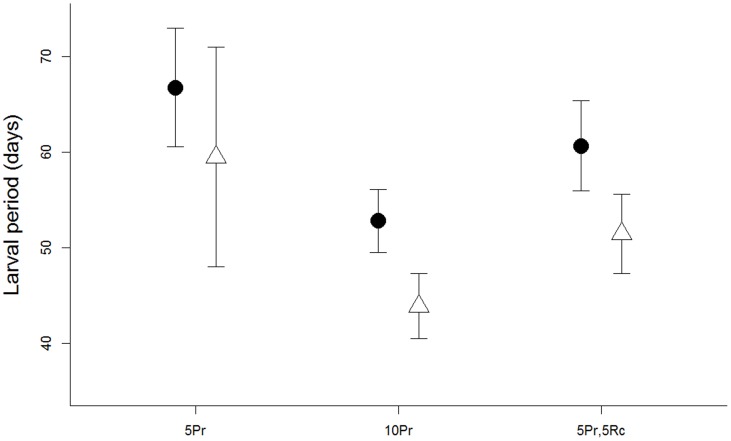
Larval period for *Pseudacris regilla* in each treatment (±SE). Treatments are indicated on the x-axis with the number of individuals and “Rc” for *Rana cascadae* and “Pr” for *P. regilla*. Closed circles represent Bd-exposed animals while open triangles represent control (unexposed) animals.

In *R. cascadae*, the proportion of individuals completing metamorphosis, pre-metamorphic survival, mass at metamorphosis and larval period were not affected by Bd exposure or species combination (p>0.10 for all comparisons). However, in *P. regilla*, exposure to Bd reduced rates of pre-metamorphic survival (*X^2^* = 5.86, p = 0.016). Additionally, larval period in *P. regilla* was affected by both Bd exposure and species combination where Bd-exposure increased the larval period (F_1,19_ = 5.56, p = 0.029; Fig. 4, Table 1). Individuals in mesocosms containing five *P. regilla* took longer to complete metamorphosis, while the 10 *P. regilla* treatment took the least time and two-species combination intermediate (F_2,19_ = 5.10, p = 0.017; Fig. 4).

## Discussion

Our results demonstrate that different amphibian species show different patterns of Bd infection across their life-stages. First, we found evidence that amphibians may lose infection as they undergo metamorphosis. At our first infection sampling (time point 1), 100% of the sampled larvae from both species were infected compared to post-metamorphic infection prevalence which was 18.18% and 81.49% for *R. cascadae* and *P. regilla*, respectively. These differences in infection at two life stages [17] indicate that the transition from the larval to post-metamorphic stages may represent an opportunity for infected individuals to shed Bd infections. Alternatively, some individuals may be able to clear their infections though time regardless of developmental progression [28]. For example, elevated temperatures can allow post-metamorphic amphibians to clear Bd infection [57], [58] so recovery from infection within a life stage is possible. In addition to differences in infection through development, we also found that species differed in their infection prevalence and severity. *P. regilla* consistently experienced higher infection severity compared to *R. cascadae* at both the larval and post-metamorphic stages (Fig. 1). Previous studies have also demonstrated similar patterns in infection [29], [47], [59]. This finding may indicate that *R. cascadae* are better at reducing or preventing infection throughout development compared to *P. regilla*. Alternatively, differences in infection after metamorphosis may be driven by infection as larvae. If *R. cascadae* are able to prevent or reduce infection as larvae, they may be less likely to re-infect themselves during metamorphosis, reducing post-metamorphic infection severity regardless of their susceptibility after metamorphosis. Since very low levels of infection may not be detected by our qPCR method, it is possible that more post-metamorphic animals were infected, but at levels too low for us to detect. However, even reducing Bd loads may help reduce the negative effects of infection [23].

Larval *P. regilla* experienced a decrease in pre-metamorphic survival when exposed to Bd compared to unexposed larvae, while *R. cascadae* survival was not influenced by Bd exposure. Since *R. cascadae* carry lower infection levels of Bd, their infections may not be severe enough to result in larval mortality. Previous studies have not found evidence for Bd-associated mortality in *P. regilla* larvae [18], [29], but these studies used relatively early stage larvae and were therefore unable to detect late-stage larval mortality, which may have occurred in this study. Keratin can be present on the tail and feet of anurans before tail resorption [17], suggesting that Bd may cause mortality before metamorphosis, even if a species is unaffected by mouthpart infection. It is possible that Bd only killed the most susceptible larval *P. regilla*, leaving more resistant individuals to complete metamorphosis. Therefore, the *P. regilla* that completed metamorphosis were likely a non-random subset of those in the mesocosms, representing individuals with higher than average resistance to Bd infection. If the susceptible individuals had survived, we may have detected even higher infection prevalence and severity in our post-metamorphic *P. regilla*.

We also found effects of density on *P. regilla* development and a trend for an effect on infection. When *P. regilla* individuals were at low densities, they had a longer larval period compared to high-density treatments (Fig. 4). Although the relationship between density and metamorphosis is complex and context-dependent [35]–[37] low larval density can slow metamorphosis when controlling for growth rates [38]. Since we did not observe differences in mass among treatments, this indicates that growth rates were not affected by our treatments. Therefore, the conditions necessary for increased larval period at low densities were present in our mesocosms. The larval period was positively correlated with infection severity (Fig. 3), indicating that a longer larval period can increase the risk of becoming infected. Thus, when *P. regilla* were maintained at low densities (five individuals versus 10), metamorphosis was slowed, lengthening the time in contact with water containing Bd. This created a pattern where low densities of *P. regilla* tended to have higher post-metamorphic infection prevalence compared to those reared at higher densities (Fig. 2). Finally, the larval period was also longer in *P. regilla* that were exposed to Bd, which suggests that infected individuals took longer to acquire the necessary resources to initiate metamorphosis due to impaired feeding behavior [13], [20].

We found evidence of pathogen spillover from *P. regilla* to *R. cascadae*, although this pattern was not significant (Fig. 2). There was a trend for the proportion of infected *R. cascadae* to be higher when combined with *P. regilla* compared to the treatments where only *R. cascadae* were present (Fig. 2). This pattern may be driven by high pathogen loads in *P. regilla* which then spill over into *R. cascadae*. We also found that Bd increased the larval period in *P. regilla* (Fig. 4) which may enhance the effectiveness of larval *P. regilla* as a reservoir for Bd in aquatic systems. Therefore, *P. regilla* may be an important driver of Bd maintenance and spread in both larval and adult hosts. Our results are consistent with previous studies [48], [49] which suggest that *P. regilla* act as reservoir species for Bd in California. In a study by Reeder et al. [49], Mountain yellow-legged frogs (*R. muscosa*) suffered population declines and local extinctions in the presence of Bd, while adult *P. regilla* persist in the same locations with high loads of Bd and demonstrated the ability to quickly shed zoospores. While the mechanisms of Bd tolerance in *P. regilla* are unknown, there are a number of physiological and behavioral mechanisms that may allow for *P. regilla* to maintain high levels of Bd infection. For example, post-metamorphic *P. regilla* exhibit patchy infections, which may allow relatively normal osmoregulation to occur in the uninfected areas [49]. More studies are necessary to understand the mechanisms behind species differences in susceptibility to Bd.

Infectious disease dynamics and development in organisms can be influenced by environmental conditions, and the conditions of the host. Our study demonstrates that infection can vary across species and across life stages. Additionally, larval conditions can influence development, which has the potential to influence infection at later life stages. Our study highlights the interacting effects of density, species combination and pathogen exposure on hosts with complex life cycles.
